# A systematic review of real-world evidence (RWE) supportive of new drug and biologic license application approvals in rare diseases

**DOI:** 10.1186/s13023-024-03111-2

**Published:** 2024-03-12

**Authors:** Shailja Vaghela, Kaniz Afroz Tanni, Geetanjoli Banerjee, Vanja Sikirica

**Affiliations:** 1 HealthEcon Consulting, Inc, Ancaster, ON Canada; 2 Harrison College of Pharmacy, Auburn University, Auburn, AL USA; 3 Moderna, Inc, Cambridge, MA USA

**Keywords:** Biologic license application, New drug application, Orphan drugs, Rare diseases, Real-world data, Real-world evidence, Systematic review, US FDA regulatory approval

## Abstract

**Background:**

Real-world evidence (RWE) generated using real-world data (RWD) presents the potential to contextualize and/or supplement traditional clinical trials for regulatory approval of rare diseases (RDs). This systematic review evaluated the use of RWD for non-oncologic RD therapies with orphan drug designation (ODD) to support efficacy outcomes in regulatory application packages to the US Food and Drug Administration (FDA). New drug applications (NDAs) and biologic license applications (BLAs) submitted between January 2017 and October 2022 were obtained from publicly available FDA drug approval websites. NDAs and BLAs of non-oncologic RD therapies were screened, and manually reviewed using RWE-related keywords. Quantitative summary of number/proportion of study types was provided, whereas qualitative synthesis focused on key categories of output assessing the use of RWD in overall drug approval process, including agency’s feedback on its strengths and key challenges.

**Results:**

A total of 868 NDAs and BLAs were identified, of which 243 were screened for non-oncologic RDs with ODD, and 151 were subsequently reviewed for the RWD used to support efficacy outcomes. Twenty (12 NDAs, 8 BLAs) applications met the review inclusion criteria. Most (19; 95%) applications used only retrospective RWD, while one (5%) collected RWD both retrospectively and prospectively. RWD studies included natural history including registry-based/retrospective historical controls (14; 70%), retrospective medical chart-reviews (4; 20%), and external RWD controls from other studies (2; 10%). The FDA generally accepted RWD studies demonstrating a large effect size despite the noted concerns and criticisms. However, the agency expressed concerns about overall quality and comparability of RWD with trial data for some applications, including RWD study designs with respect to differences in patients’ baseline characteristics, missing information, and potential bias and measurement errors.

**Conclusions:**

This systematic review highlights potential benefits of appropriately conducted RWE studies in RD, which can strengthen the clinical evidence for efficacy comparison and contextualization to support product approval efforts, particularly when a large magnitude of effect is observed for the new intervention. Nonetheless, quality and completeness of RWD and its comparability with trial data remain areas of concern that can serve as valuable learnings for advancing future science and regulatory approvals.

**Supplementary Information:**

The online version contains supplementary material available at 10.1186/s13023-024-03111-2.

## Background

Real world evidence (RWE) is defined as the clinical evidence derived from real world data (RWD), which reflects a patient’s health state and/or delivery of healthcare. The RWD is collected from various sources such as electronic health records (EHR), medical/pharmacy claims and billing activities, product and disease registries, cross sectional surveys [1]. RWE has the potential to contextualize and/or supplement traditional randomized clinical trials (RCTs) for obtaining regulatory approval for therapies [1–4]. This is particularly important for non-oncologic therapies targeting rare diseases (RDs) and seeking orphan indications [5]. The Orphan Drug Act defines a rare disease or condition as one [a] that affects fewer than 200,000 persons in the United States (US) or [b] for which there is no reasonable expectation that the cost of developing a drug and making it available in the US will be recovered from sales in the country [6]. For RDs, generating robust clinical evidence can be challenging due to small patient populations, limited knowledge of the disease’s natural history, or the impracticality of conducting an RCT due to patient feasibility or ethical considerations [7].

Although the use of RWE in regulatory decision-making is not yet universally accepted, opinions within regulatory agencies are evolving; and there is a growing recognition to consider RWE in the drug approval process. Various authorities, including regulatory bodies such as the Food and Drug Administration (FDA) in the US [4] and the European medicine Agency (EMA) [8], health technology assessment bodies [e.g., National Institute for Health and Care Excellence (NICE) in the United Kingdom (UK) [9] and Canadian Agency for Drugs and Technologies in Health (CADTH) in Canada [10]], as well as international organizations such as the International Society for Pharmacoeconomics and Outcomes Research (ISPOR) and the International Society for Pharmacoepidemiology joint special task force have published guidance on using RWE in the drug submission process [11]. In the US, the 21st Century Cures Act was enacted on December 13, 2016, to expedite medical product development and deliver novel therapies to patients efficiently. It also broadened the application of RWE by the US FDA, expanding its use beyond post-market surveillance and recognizing its potential to inform regulatory decision-making throughout drug development [4, 12].

Despite the FDA’s willingness to accept RWE in drug approval assessment, there remains a lack of knowledge among stakeholders regarding the agency’s acceptance of RWE for certain orphan indication approvals. While a limited number of prior published studies have reviewed the FDA’s stance on RWE for safety and efficacy in the context of New Drug Applications (NDAs) and Biologic License Applications (BLAs) [13–16], no prior research has explicitly focused on RD therapies and their reliance on RWE to support efficacy outcomes. To address this gap, a systematic literature review (SLR) of NDAs and BLAs submitted to the FDA Post 21st Century Cures Act, was conducted aiming to evaluate the use of RWE in support of efficacy outcomes and approval within regulatory submissions for RD therapies.

## Methods

### Search strategy and eligibility criteria

In this systematic review, all NDAs and BLAs submitted between January 2017 and October 2022 were identified and their submission packages were obtained from publicly available FDA drug approval bodies- Center for Drug Evaluation and Research (CDER) [17], and Center for Biologics Evaluation and Research (CBER) [18]. The lists of approved drugs and biologics were screened for the review classifications with ‘orphan drug designation (ODD)’. Subsequently, applications approved for oncologic indications were excluded to solely target RD therapies. The corresponding FDA review documents of RD therapies including clinical-, integrated-, multidisciplinary-, and statistical reviews, respectively were retrieved. These documents were manually searched for RWE keywords including ‘chart abstraction’, ‘chart review’, ‘claims’, ‘electronic medical record’, ‘external control’, ‘historical control’, ‘medical chart review’, ‘medical record’, ‘natural history’, ‘non-experimental’, ‘non-interventional’, ‘observational’, ‘pragmatic clinical trial’, ‘real world’, ‘registry’. The full text reports were reviewed to identify applications that incorporated RWD in their regulatory submission package. Finally, the NDAs and BLAs for non-oncologic RD therapies with ODD status utilizing RWD to support efficacy outcomes, whether for contextualization or comparison with clinical trial data, were included in the review. Applications using RWD for patient recruitment, safety analysis, or prospective post-marketing surveillance registry/data plans etc. were excluded. Two authors independently conducted screening and full-text reviews, while discrepancies were resolved through collaborative discussions among all authors.

### Data extraction and synthesis

For the qualitative synthesis, three categories of elements were extracted from each of the NDAs and BLAs included: (A) Application characteristics pertaining to therapy, indication, epidemiology, type of review and approval dates; (B) details on pivotal studies for application; and (C) RWE information including RWD study approach, methods and designs, FDA feedback and RWD details in the FDA label claims sought by the Sponsors.

One author manually performed data extraction using a predefined form developed in Microsoft® Excel. To extract the elements of the above-mentioned three categories, full reports were reviewed section-wise without relying solely on key terms for variables of interest. Another author conducted a quality check for all the extracted data by reviewing the respective sections of the full text reports from the submission packages.

A quantitative summary detailing the number and proportion of study types was provided, while the qualitative synthesis was focused on determining the role of the RWE in the overall drug approval process, identifying the key challenges limiting the usefulness of the RWD, and examining the features of RWD studies that strengthened the outcomes of the review.

## Results

**Fig. 1:**
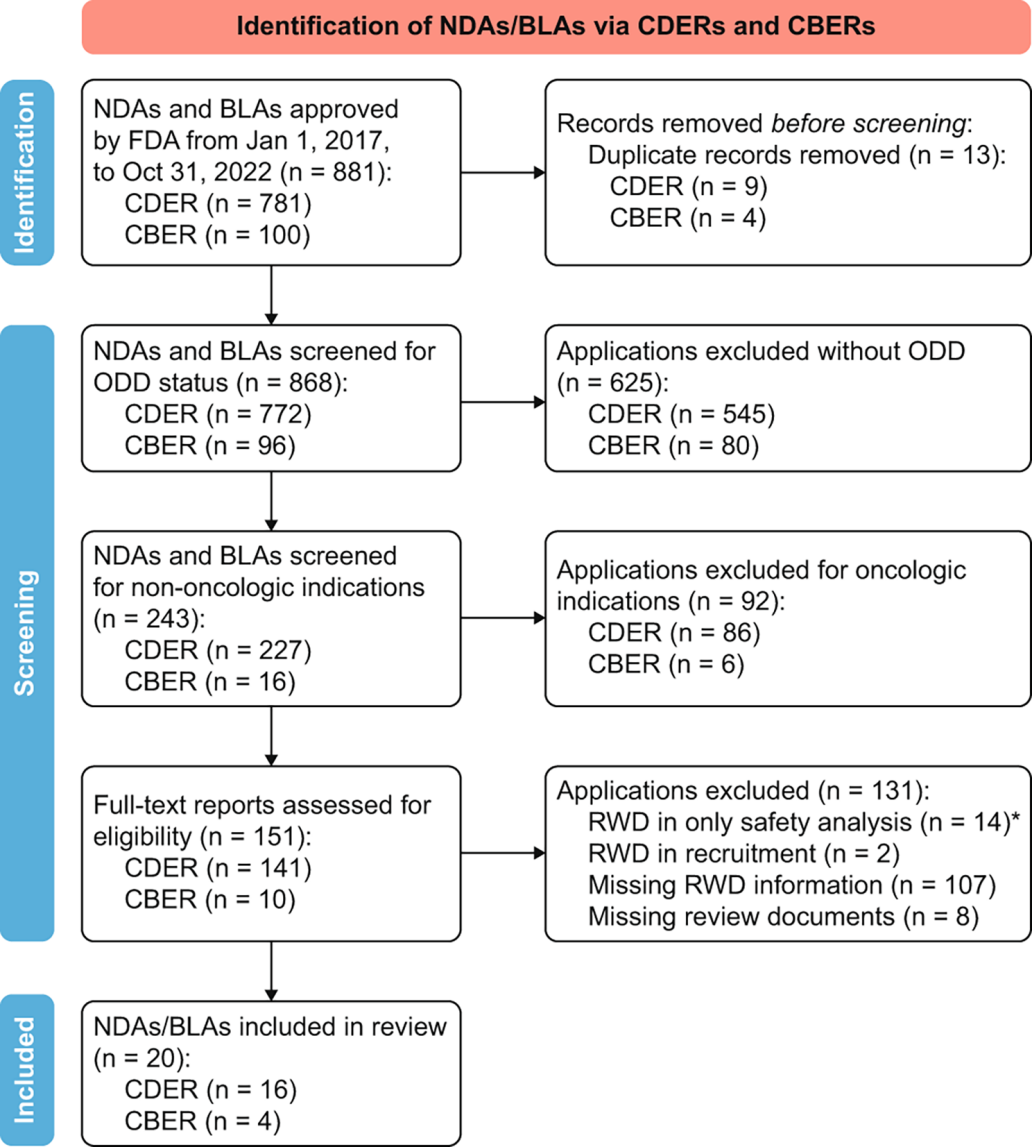
PRISMA diagram of inclusion and exclusion of NDAs and BLAs in the systematic review. Abbreviations: BLA, Biologic license application; CBER, Center for Biologics Evaluation and Research; CDER, Center for Drug Evaluation and Research; FDA, Food and Drug Administration,USA; n, Number of approvals; NDA, New drug application; ODD, Orphan drug designation; RWD, Real-world data. *Number of applications with the use of RWD for safety analysis were excluded, wherein RWD was used for prospective, post-marketing safety registry/data plans etc. *Note: PRISMA diagram template was adapted from Page MJ, McKenzie JE, Bossuyt PM, Boutron I, Hoffmann TC, Mulrow CD, et al. The PRISMA 2020 statement: an updated guideline for reporting systematic reviews. BMJ 2021;372:n71. doi*: 10.1136/bmj.n71

A total of 868 applications (772 NDAs, 96 BLAs) approved by the FDA between January 1, 2017 and October 31, 2022, were identified [17–19]. Of these, 243 applications were screened for orphan drug designation (ODD) status and non-oncology RD indications, and 151 applications with full text were subsequently reviewed for RWD supporting efficacy outcomes. Finally, 20 (12 NDAs, 8 BLAs) applications for RD therapies with ODD were included in the review. A detailed PRISMA diagram illustrating the inclusion and exclusion process is depicted in Fig. 1.

Seventeen applications (85%) went through priority reviews, while the remaining three (15%) applications were subject to standard reviews. Three (15%) therapies, namely elivaldogene autotemcel, viltolarsen and vosoritide, received accelerated approvals. Notably, six (30%) of the approved medications were indicated for neuromuscular and bone-related disorders, while five (25%) were approved for rare metabolic disorders. A summary of the application characteristics is outlined in Table 1.

**Table 1:** Application characteristics of NDAs and BLAs included in the systematic review

Therapy– Generic (US Brand name)	Approved Indication	Epidemiology of Disease	NDA/BLA	Review Type	Approval date
Elivaldogene autotemcel (Skysona) [20]	Early cerebral adrenoleukodystrophy (CALD)	US prevalence: 35–40% of the 1:20,000 males affected with X-ALD	BLA	Priority	Sept 16, 2022*
Vutrisiran (Amvuttra) [21]	Polyneuropathy (PN) of hereditary transthyretin mediated (hATTR) amyloidosis in adults	US incidence: 1/100,000 in U.S. Caucasians; US prevalence of hATTR-PN: 100 to 2,500 individuals	NDA	Standard	Jun 13, 2022
Vosoritide (Voxzogo) [22]	Increase in linear growth in children with achondroplasia ages 5 and older with open epiphyses	Global incidence: 1 in 25,000 births	NDA	Priority	Nov 19, 2021*
Allogeneic processed thymus tissue- agdc (Rethymic) [23]	Immune reconstitution in pediatric patients with congenital athymia	US incidence: 20 newborns p.a.	BLA	Priority	Oct 8, 2021
Fosdenopterin (Nulibry) [24]	Molybdenum cofactor deficiency (MoCD) type A	US prevalence: 45 to 54 patients, all under 10 years of age; US incidence: 0.24–0.29 per 100,000 infants [25]	NDA	Priority	Feb 26, 2021
Lonafarnib (Zokinvy) [26]	Hutchinson-Gilford progeria syndrome (HGPS) and processing deficient progeroid laminopathies (PL)	HGPS Global incidence: 1 in 4 million births; Global prevalence: 1 in 20 million PL US prevalence: 1 in 25 million.	NDA	Priority	Nov 20, 2020
Viltolarsen (Viltepso) [27]	Duchenne muscular dystrophy	Global incidence: 1 in 5,000 live male births; US prevalence: 16 cases per 100,000 live male births	NDA	Priority	Aug 12, 2020*
Risdiplam (Evrysdi) [28]	Spinal muscular atrophy	Global incidence: 8.5 to 10.3 per 100,000 live births	NDA	Priority	Aug 7, 2020
Triheptanoin (Dojolvi) [29]	A source of calories and fatty acids in the treatment of long-chain fatty acid oxidation disorders (LC-FAOD)	US, Germany, Australia incidence: 1 in 9,300 individuals	NDA	Standard	Jun 30, 2020
Pretomanid Tablet (Pretomanid) [30]	Pulmonary extensively drug-resistant (XDR) and treatment-intolerant/nonresponsive (TI/NR) multidrug-resistant (MDR) tuberculosis in adults	US reported cases in 2020 [31]: MDR-TB: 56 cases; XDR TB: 1 case	NDA	Priority	Aug 14, 2019
Onasemnogene abeparvovec-xioi (Zolgensma) [32]	Spinal muscular atrophy (SMA)	SMA type 1 global incidence: 1 in 10,000 live births; Global prevalence: 1–2 per 100,000	BLA	Priority	May 24, 2019
Triclabendazole (Egaten) [33]	Fascioliasis	Global prevalence: 2.4 to 17 million individuals	NDA	Priority	Feb 13, 2019
Stiripentol (Diacomit) [34]	Dravet syndrome	US incidence: 1 in 40,000 infants [35]	NDA	Priority	Aug 20, 2018
Fish oil triglycerides inj. Emulsion (Omegaven) [36]	Parenteral nutrition-associated cholestasis (PNAC)	Global incidence: 28.2% In children who received PN for > = 14 days	NDA	Priority	Jul 27, 2018
Burosumab (Crysvita) [37]	X-linked hypophosphatemia (XLH)	Global incidence: 1 in 20,000 live births; US prevalence: 3,000 pediatric and 12,000 adult patients	BLA	Priority	Apr 17, 2018
Voretigene neparvovec (Luxturna) [38]	Biallelic RPE65 mutation-associated retinal dystrophy	US prevalence: 1,000 to 2,000 individuals [39]	BLA	Priority	Dec 19, 2017
Emicizumab-kxwh (Hemlibra) [40]	Hemophilia A (congenital factor VIII deficiency)	US incidence:20,000 live births; Global incidence: 400,000 live births	BLA	Priority	Nov 16, 2017
Vestronidase alfa-vjbk (Mepsevii) [41]	Mucopolysaccharidosis type 7 (MPS VII)	Global prevalence: 1 in 250,000	BLA	Priority	Nov 15, 2017
Cerliponase alfa (Brineura) [42]	Neuronal ceroid lipofuscinosis type 2 (CLN2)	US & Europe incidence: 0.56-4 pts per 100,000 live births	BLA	Priority	Apr 27, 2017
Thiotepa (Tepadina) [43]	Class 3 β-thalassemia	Global incidence: 100,000 children p.a.	NDA	Standard	Jan 26, 2017

BLA, biologic license application; NDA, new Drug Application; US, United States

*Accelerated approval

Table 2 describes the characteristics of the actual RWD included in the FDA review packages. Most (19; 95%) applications utilized a retrospective approach to gather RWD, whereas only fosdenopterin (for molybdenum cofactor deficiency [MoCD] type A, collected natural history data both retrospectively and prospectively [24]. Three (15%) applications used RWD for contextualization, 10 (50%) used it for comparison to the drug or biologic seeking approval, while seven (35%) applications used RWD for both contextualization as well as comparison.

**Table 2:** Characteristics of real-world data (RWD) used in FDA review packages

Therapy– Generic (US Brand name)	Approved Indication	RWD Study Design	Temporality: Retrospective/ Prospective/ Both	Purpose: Contextualization/ Comparison/ Both	Main Objective(s)	RWD Study sample size	Duration matches Trial	A-Priori Protocol	Eligibility Criteria Matched	Methods for Bias OR Missing Data Reported
Elivaldogene autotemcel (Skysona) [20]	Early cerebral adrenoleukodystrophy (CALD)	Natural History	Retrospective	Both	- Evaluate natural history of untreated CALD - Evaluate efficacy/safety of alloHSCT in CALD	172	✗	✓	✓	✗
Vutrisiran (Amvuttra) [21]	Polyneuropathy (PN) of hereditary transthyretin mediated (hATTR) amyloidosis in adults	External Placebo Control	Retrospective	Comparison	- Determine efficacy of vutrisiran in pts. with hATTR amyloidosis by evaluating effect on neurologic impairment compared to external controls	77	✓	✓	✓	✓
Vosoritide (Voxzogo) [22]	Increase in linear growth in children with achondroplasia ages 5 and older with open epiphyses	Natural History	Retrospective	Comparison	- Demonstrate long term safety and efficacy	Matched at baseline: 559; Matched at 5-year: 360	✓	✓	✓	✓
Allogeneic processed thymus tissue- agdc (Rethymic) [23]	Immune reconstitution in pediatric patients with congenital athymia	Natural History	Retrospective	Comparison	- Assess survival at year 1 and 2	49	✓	✓	✓	Bias: ✓ Missing data: ✗
Fosdenopterin (Nulibry) [24]	Molybdenum cofactor deficiency (MoCD) type A	Natural History	Both	Both	- Characterize natural history of MoCD Type A - Observe clinical outcomes, including survival	Retro: 37 Prosp:14	✓	✓	✓	Bias: ✓ Missing data: ✗
Lonafarnib (Zokinvy) [26]	Hutchinson-Gilford progeria syndrome (HGPS) and processing deficient progeroid laminopathies (PL)	Registry-based natural history	Retrospective	Comparison	- Evaluate reduced mortality in patients with HGPS compared to matched controls	Unmatched: 81; Matched: 62 (1:1)	✗	✗	✓	Bias: ✓ Missing data: ✗
Viltolarsen (Viltepso) [27]	Duchenne muscular dystrophy	Natural History	Retrospective	Comparison	- Compare functional endpoints	69	✓	NR	✓	Bias: ✓ Missing data: ✗
Risdiplam (Evrysdi) [28]	Spinal muscular atrophy	Natural History	Retrospective	Both	- Compare proportion of patients sitting without support	NR	NR	NR	✓	✗
Triheptanoin (Dojolvi) [29]	A source of calories and fatty acids in the treatment of long-chain fatty acid oxidation disorders (LC-FAOD)	Medical Chart Review	Retrospective	Comparison	- Historical medical record review to compare major clinical event rates	29	CL201:✓ CL202:✗	✗	✓	✗
Pretomanid Tablet (Pretomanid) [30]	Pulmonary extensively drug-resistant (XDR) and treatment-intolerant/nonresponsive (TI/NR) multidrug-resistant (MDR) tuberculosis in adults	Historical Control	Retrospective	Both	- Provide contextualization for efficacy rates and compare with historical control	202	✗	✓	✓	Bias: ✓ Missing data: ✗
Onasemnogene abeparvovec-xioi (Zolgensma) [32]	Spinal muscular atrophy (SMA)	Natural History	Retrospective	Comparison	- To compare survival at 14 months of age; and the proportion of subjects able to sit independently for = 30 s by 18 months of age.	23	NR	✓	✓	✗
Triclabendazole (Egaten) [33]	Fascioliasis	Historical Control	Retrospective	Comparison	- Compare cure rates	37	✓	✓	NR	✗
Stiripentol (Diacomit) [34]	Dravet syndrome	Medical Chart Review	Retrospective	Both	- Historical chart review from 2002–2012 to compare benefits/effects	29	✗	✗	✓	✗
Fish oil triglycerides inj. Emulsion (Omegaven) [36]	Parenteral nutrition-associated cholestasis (PNAC)	Natural History	Retrospective	Both	- Evaluate growth of pediatric patients with PNAC on Omegaven or Intralipid; - Compare the effect and safety	Study 34: PP– 52; PM– 26 Study 35: PP– 24; PM– 15	✓	✓	✓	✓
Burosumab (Crysvita) [37]	X-linked hypophosphatemia (XLH)	Natural History	Retrospective	Both	- Characterize change in rickets severity over time with conventional therapy; - Comparison to uncontrolled ph II study	52	✓	✓	✓	Bias: ✓ Missing data: ✗
Voretigene neparvovec (Luxturna) [38]	Biallelic RPE65 mutation-associated retinal dystrophy	Medical Chart Review	Retrospective	Contextualization	- Evaluate the disease condition	70	✓	✗	✓	✗
Emicizumab-kxwh (Hemlibra) [40]	Hemophilia A (congenital factor VIII deficiency)	External Control	Retrospective	Contextualization	- Document number, types, and treatment of bleeds under routine clinical practice	Cohort A: 103; Cohort B: 24; Cohort C: NR	NR	✓	NR	✗
Vestronidase alfa-vjbk (Mepsevii) [41]	Mucopolysaccharidosis type 7 (MPS VII)	CL001: Medical Chart Review; CL002: Patient Survey	CL001: Retrospective; CL002: Retrospective history data	Contextualization	- Evaluate impact on clinical outcomes, - Identify the type and severity of clinical symptoms; impact of symptoms on functional status; inform endpoint selection for clinical studies	Study CL001: 50; Study CL002: 10	NR	NR	✓	✗
Cerliponase alfa (Brineura) [42]	Neuronal ceroid lipofuscinosis type 2 (CLN2)	Registry-based natural history	Retrospective	Comparison	- Compare efficacy endpoints	69	✓	✓	✓	✗
Thiotepa (Tepadina) [43]	Class 3 β-thalassemia	Historical Control	Retrospective	Comparison	- Compare incidence of graft rejection following allogeneic BMT with control arm	71	✓	✓	✓	✗

✗, no; ✓, yes; BMT, bone marrow transplant; CALD, cerebral adrenoleukodystrophy; hATTR, hereditary transthyretin mediated; HGPS, Hutchinson-Gilford progeria syndrome; HSCT, hematopoietic stem cell transplant; MoCD, molybdenum cofactor deficiency; MPS VII, mucopolysaccharidosis type 7; NR, not reported; PL, progeroid laminopathies; PNAC, parenteral nutrition-associated cholestasis; RWD, real-world data

Of the 20 applications, 12 (60%) applications were found to match the duration of RWD with the duration of pivotal clinical trials. Furthermore, 13 (65%) applications had an a priori protocol in place as the FDA highly recommends prior discussion of protocol and study design development with the agency. Seventeen (85%) applications were reported to match patient eligibility criteria, however, the FDA commented on differences in patient population and/or missing information on key elements for 10 (50%) applications. All applications (20; 100%) reported sample sizes for RWD studies, which varied mainly depending on the prevalence and rareness of the disease; ranging from 10 in Study CL002 of vestronidase alfa-vjbk for mucopolysaccharidosis type 7 (MPS VII) [41] to 559 in the matched natural history cohort at baseline for vosoritide (for achondroplasia) [22]. Only three (15%) applications– vutrisiran (for the treatment of the polyneuropathy of hereditary transthyretin mediated [hATTR] amyloidosis in adults) [21], vosoritide (for achondroplasia) [22] and fish oil triglycerides injection emulsion (for parenteral nutrition-associated cholestasis [PNAC] [36])– reported methods for handling biases and missing data, whereas 17 applications (85%) did not report any methods for handling missing data, and five (25%) reported methods for handling only bias. Some of the key approaches to reduce selection or detection biases included matching algorithms for key attributes, centralized site monitoring, third-party data collection and blinded reading for trial results [22, 24, 37]. While for missing data, prespecified imputation plan were included (e.g., for vutrisiran [21] and vosoritide [22]).

Table 3 outlines FDA’s feedback and RWD reported in their label claims and details on clinical trial studies for the reviewed applications are presented in Additional File 1.

**Table 3:** FDA feedback on submissions and RWD reported in FDA labels

Therapy– Generic (US Brand name)	Approved Indication	NDA/BLAs’ Primary Clinical Studies^a^	RWD Study Design	FDA Feedback on RWD	RWD reported in FDA Label Claim (Y/N)
Elivaldogene autotemcel (Skysona) [20]	Early cerebral adrenoleukodystrophy (CALD)	1 nonrandomized, open label, single arm study	Natural History	- Overall population not comparable to trial population - Potential selection bias and missing data - Potentially subjective elements of definitions	✓
Vutrisiran (Amvuttra) [21]	Polyneuropathy (PN) of hereditary transthyretin mediated (hATTR) amyloidosis in adults	1 open label RCT with external placebo control (RWD)	External Placebo Control	- Notable differences in baseline pt. characteristics and disease severity compared to trial - Large effect size was sufficient to overcome potential biases and support efficacy outcomes	✓
Vosoritide (Voxzogo) [22]	Increase in linear growth in children with achondroplasia ages 5 and older with open epiphyses	3 clinical studies: 1 RCT; 1 open label, single arm; 1 long-term efficacy/safety	Natural History	- Limited data on genetic diagnosis, medical history, medications; but unlikely to skew results in favor of vosoritide - Measurement errors were not expected to have a significant impact on analyses	✗
Allogeneic processed thymus tissue- agdc (Rethymic) [23]	Immune reconstitution in pediatric patients with congenital athymia	Efficacy data derived from 7 open label, non-randomized studies	Natural History	- Missing information on phenotypes, underlying genetic defects, co-morbidities, supportive care - Consistent large survival effects, with a favorable benefit risk profile in patients	✓
Fosdenopterin (Nulibry) [24]	Molybdenum cofactor deficiency (MoCD) type A	2 open label, single arm studies	Natural History	- Potential for selection bias was adequately overcome; Detection bias didn’t impact observed survival benefit	✓
Lonafarnib (Zokinvy) [26]	Hutchinson-Gilford progeria syndrome (HGPS) and processing deficient progeroid laminopathies (PL)	2 open label, single arm studies	Registry-based natural history	- Differences in number of patients among cohorts; Treated cohort had a substantially higher censoring rate over time than the matched untreated cohorts - Missing data on concomitant cardiovascular medications in control arm	✓
Viltolarsen (Viltepso) [27]	Duchenne muscular dystrophy	1 double blind, placebo and NH controlled study	Natural History	- Heterogeneity of the disease, patient characteristics, care - Imprecision of population matching due to lack of control of all known and unknown biases	✗
Risdiplam (Evrysdi) [28]	Spinal muscular atrophy	2 studies: 1 RCT, 1 open label, single arm study	Natural History	- Considers the external natural history control as sufficient - NH of spinal muscular atrophy is well understood	✓
Triheptanoin (Dojolvi) [29]	A source of calories and fatty acids in the treatment of long-chain fatty acid oxidation disorders (LC-FAOD)	3 studies: 1 randomized parallel design; 2 open label, single arm studies	Medical Chart Review	- Heterogeneity in disease severity, dietary management, data collection of lab and major clinical events, - Dietary details missing for many patients, prior treatment history not properly collected or accounted for in analysis	✗
Pretomanid Tablet (Pretomanid) [30]	Pulmonary extensively drug-resistant (XDR) and treatment-intolerant/nonresponsive (TI/NR) multidrug-resistant (MDR) tuberculosis in adults	1 single arm study	Historical Control	- Trial patients had much greater rates of treatment success and lower mortality rates compared to historical control	✓^b^
Onasemnogene abeparvovec-xioi (Zolgensma) [32]	Spinal muscular atrophy (SMA)	1 open label, single arm study	Natural History	- NH results indicated that the expected treatment effect is large, readily ascertained, and shows close temporal association with the intervention	✓
Triclabendazole (Egaten) [33]	Fascioliasis	2 open label, randomized studies	Historical Control	- Large treatment effect was observed comparing with the historical control	✗
Stiripentol (Diacomit) [34]	Dravet syndrome	2 placebo-controlled RCTs	Medical Chart Review	- Methods are not powered to detect significant effects	✗
Fish oil triglycerides inj. Emulsion (Omegaven) [36]	Parenteral nutrition-associated cholestasis (PNAC)	2 open label studies	Natural History	- Covariate measurement errors, unmet model assumptions, biases in endpoint estimates	✓
Burosumab (Crysvita) [37]	X-linked hypophosphatemia (XLH)	2 open label studies	Natural History	- NH study and trial results provide support for the effectiveness of burosumab therapy	✗
Voretigene neparvovec (Luxturna) [38]	Biallelic RPE65 mutation-associated retinal dystrophy	1 open label RCT with cross over design	Medical Chart Review	- Chart review contextualized natural history of retinal dystrophy including many mutations and a variety of clinical diagnoses	✗
Emicizumab-kxwh (Hemlibra) [40]	Hemophilia A (congenital factor VIII deficiency)	2 studies: 1 randomized, open label with 2 non- randomized arms; 1 open label, single arm study	External Control	- Superiority over other products has not been proven, and results should be interpreted with caution - Note: RWD was used only for contextualization	✗
Vestronidase alfa-vjbk (Mepsevii) [41]	Mucopolysaccharidosis type 7 (MPS VII)	1 placebo controlled RCT	Medical Chart Review	- No clinical examinations were completed, and degree of cognitive disability appeared to be underestimated in non-interventional studies, which could impact endpoint selection, completion, and interpretation	✗
Cerliponase alfa (Brineura) [42]	Neuronal ceroid lipofuscinosis type 2 (CLN2)	1 open label, single arm study	Registry-based natural history	- Differences in patient characteristics and clinician-reported outcomes used to compare disease progression in both arms	✓
Thiotepa (Tepadina) [43]	Class 3 β-thalassemia	1 retrospective, observational, study for efficacy assessment	Historical Control	- Patient demographics were generally similar at baseline, except for history of splenectomy - Justifiable study design as an RCT could not be blinded and enrolment would be impractical due to rareness of the disease	✓

✗, no; ✓, yes; NH, natural history; RCT, randomized clinical trial; RWD, real-world data,

^a^Details on primary clinical studies for the reviewed applications are presented in Additional File 1

^b^Mentioned that “the success rate significantly exceeded the historical success rates for extensively drug-resistant tuberculosis (XDR-TB) based on a literature review”, without providing details on rates for historical arm or review

In total, nine out of the twenty applications (45%) received positive feedback from the FDA regarding the utilization of RWD. The favorable response was attributed to various factors, including a significantly large effect size observed in the analysis, the appropriateness and justifiability of the RWD design, and the incorporation of RWD as external controls for comparison or contextualization in the studies. However, the FDA expressed concerns about the implementation of RWD in eleven applications (55%). The concerns mainly revolved around several key aspects, such as differences in baseline characteristics of patient populations of the RWD and clinical trial(s), imprecise population matching techniques, insufficient information on key input elements, the presence of potentially subjective elements in defining study endpoints, and the possibility of selection bias and measurement error.

The majority of the reviewed RD applications used retrospective historical cohort study data (3 applications) or natural history study control data (8 retrospective; 1 prospective and retrospective). Of these 12 applications, five (42%) received FDA’s positive feedback and their FDA label claims also reported the use of natural history data. The FDA accepted their justification for using natural history data/historical controls, and/or notable large effect size with the potential to overcome selection bias or measurement error. Three applications– elivaldogene autotemcel (for early cerebral adrenoleukodystrophy [CALD]) [20], pretomanid tablets (for pulmonary extensively drug-resistant [XDR] and treatment-intolerant/nonresponsive [TI/NR] multidrug-resistant [MDR] tuberculosis in adults) [30], and fish oil triglycerides injection emulsion (for the treatment of PNAC) [36]– received criticism for differences in population characteristics and endpoint definitions, and potential biases and measurement errors. Despite these shortcomings, all three applications reported RWD in their label claim. In contrast, two applications– triclabendazole (for fascioliasis) [33] and burosumab (for X-linked hypophosphatemia [XLH]) [37]– received positive feedback on their RWD study design and effect size in the review documents, however, RWD was not reported in the label claims for both the drugs. Finally, viltolarsen (for Duchenne muscular dystrophy [DMD]) [27] faced criticism for the use of RWD due to the heterogeneity of the disease and patient characteristics, and lack of controlling biases, and consequently, the use of RWD was excluded from its label claim.

Two applications (10%)– lonafarnib (for Hutchinson-Gilford Progeria syndrome [HGPS] and processing deficient progeroid laminopathies) [26] and cerliponase alfa (for neuronal ceroid lipofuscinosis type 2 [CLN2]) [42]– used registry-based natural history data/cohorts for comparison. Despite of criticism on differences in patient characteristics, censoring rates or use of different versions of ClinROs, both the drugs reported RWD in their label claims when compared to their respective single-arm drugs or biologics from their pivotal open label trials.

Two applications (10%)– vutrisiran (for the polyneuropathy of hATTR amyloidosis in adults) [21] and emicizumab-kxwh (for hemophilia A [congenital factor VIII deficiency] with factor VIII inhibitors) [40]– used external RWD controls from other studies for comparison and contextualization, respectively. The HELIOS-A, a phase III open label study for vutrisiran, used a placebo group of the APOLLO (ALN-TTR02-004) study as an external RWD placebo control. Given the life-threatening nature of hATTR amyloidosis and the existence of approved therapies, it would not be ethical to use a concurrent placebo control group, and hence the FDA deemed this approach reasonable [21]. It had a large effect size despite notable differences in patient characteristics and hence it reported RWD in the label claim. Conversely, emicizumab-kxwh used RWD for contextualization only and did not report any RWD in the label claim as superior efficacy over other products was not proven.

Four applications (20%)– triheptanoin (approved for a source of calories and fatty acids in the treatment of long-chain fatty acid oxidation disorders [LC-FAOD]) [29], stiripentol (for Dravet syndrome) [34], voretigene neparvovec (for biallelic RPE65 mutation-associated retinal dystrophy) [38], and vestronidase alfa-vjbk (for MPS VII) [41]– utilized retrospective medical chart reviews for RWD. However, the FDA criticized all these applications for various reasons such as differences in patient characteristics, missing information, inadequate power for methods to detect effect size, or impact on endpoint selection. Consequently, none of these applications reported RWD in their label claims.

## Discussion

As there is a growing recognition of potential utility of RWE in drug approval process, our systematic review provided a comprehensive synthesis of RWD utilization in supporting efficacy outcomes for RD applications submitted to the FDA since the implementation of the 21st Century Cure Act. Our review provided a detailed analysis of the applications employing RWE, the key aspects of RWD submitted, as well as the impact on FDA decision-making and inclusion as part of the approval.

The FDA evaluated the appropriateness and quality of RWD study designs in the reviewed applications, regardless of the specific study design utilized. Of the 20 applications in rare disease, nine (45%) received overall positive feedback from the FDA on RWD, attributed to a significantly large effect size, justifiable RWD design, and/or the use of external controls for comparison or contextualization [21–24, 28, 32, 33, 37, 43]. Despite variances in baseline characteristics and potential biases, these challenges seemed mitigated by the significantly large effect size observed in single-arm comparators. Furthermore, of these applications with favorable feedback, six approvals (five natural history/historical controls; one external placebo control) also reported RWD for contextualization and/or efficacy comparison in their FDA label claims [21, 23, 24, 28, 32, 43], which potentially indicates the FDA’s acceptance of the comparability of RWD generated to compare with that generated from clinical trials. This acceptance was particularly noteworthy in applications where the effect size was sufficiently large to overcome potential biases or measurement errors. Additionally, the FDA was more receptive to RWD when blinding in RCT was not feasible, and enrollment was either difficult or impractical due to the rareness of the disease.

The agency criticized RWD in 11 (55%) applications, citing concerns about differences in patient population, potential selection bias, measurement errors, imprecision of population matching, missing information on key input elements, or potentially subjective elements of study endpoint definitions. Of these applications, six did not report RWD in their label claims, whereas five applications reported RWD in their label claims despite some of the aforementioned issues. For instance, the FDA commented on cerliponase alfa for differences in patient characteristics between the single arm RCT and registry-based cohort, as well as the use of a different version of the ClinRO in both arms. Nevertheless, the FDA label claim reported results for the comparative efficacy analysis on the motor domain scale for the indication of neuronal ceroid lipofuscinosis type 2 (CLN2) [42].

This systematic review focused on the use of RWD for contextualization and/or comparison for efficacy outcomes in RD therapies. Arondekar et al. conducted somewhat a similar review for only oncology applications, however, they did not include a comparison of FDA’s feedback on oncology applications with the inclusion of RWD in label claims [13]. Purpura et al. performed a review to quantify how many approved applications incorporated RWE in any form (i.e., for safety or efficacy) from January 2019 to June 2021 [14]. Izem et al. reviewed RWD for only contextualization in oncology and RD applications since 2000 and discussed some of the approvals as case studies [15]. Similarly, Seifu et al. assessed RWD for effectiveness in any indication and evaluated three applications in detail as case studies [16]. Our study, with a different approach from prior studies and focusing explicitly on efficacy outcomes for RD therapies, further consolidated the growing importance of RWE in drug approval process for RDs. The FDA has released a guidance on the use of RWD sources and best methodological practice [44]. Hence, our study also helps researchers and developers in enhancing their understanding of the science and the specific elements that the FDA is willing to accept or reject within RWD studies for RDs. The findings of this study should, however, be interpreted in the context of certain limitations.

This SLR was restricted to publicly available information on non-oncological RD applications approved by the FDA at the time of the study, and solely captured the FDA’s feedback provided in the review documents of rare disease orphan designation submissions. Hence, the study findings may not be comprehensive and generalizable to other indications/applications within the FDA. Furthermore, the viewpoints of other regulatory and health technology authorities may differ as well.

Additionally, the FDA’s review process varied by application, which was mainly influenced by the rareness of disease, rationale for RWD usage, quality of RWE study design components and other such contributing factors. Hence, it was not possible to draw a definitive pattern to strongly recommend RWD design methodology for RD regulatory submissions. Instead, this review provided key themes and considerations that should be considered when generating RWE for regulatory and HTA submissions for RDs. Moreover, as reviewed applications for RD therapies had used different types of studies to generate RWD, further research is warranted to focus on each theme and provide a detailed synthesis and roadmap with useful recommendations to enhance scientific validity.

## Conclusions

This systematic review explored the utilization of RWD supporting efficacy outcomes in non-oncologic RD applications, revealing general acceptance for those with a significantly large effect size. Despite acceptance, the FDA expressed concerns about RWD study designs, emphasizing issues like differences in baseline characteristics of the population, imprecision of population matching, handling missing information or potential selection bias and measurement error. This review serves to inform future researchers and applicants with insights into the FDA’s comments and concerns regarding the use of RWD in regulatory submissions. It highlights key areas for improving the RWD to appropriately contextualize and compare it with clinical trial populations, to derive the unbiased effect size of intervention, and to appropriately support evidence packages in regulatory submissions. With the increasing use of RWD in regulatory applications, there is an opportunity to enhance both the understanding of FDA’s expectations for utility and quality of RWD, as well as the applicants’ adherence to such expectations.

### Electronic supplementary material

Below is the link to the electronic supplementary material.

**Additional file 1:** Clinical studies. Details of clinical studies associated with new drug and biologic license applications in rare diseases that contain real-world data.

## Data Availability

The article used data from publicly available regulatory review documents from the US Food and Drug Administration website- https://www.accessdata.fda.gov/scripts/cder/daf/index.cfm.
